# Characterization of a bronchoscopically induced transgenic lung cancer pig model for human translatability

**DOI:** 10.1038/s41684-025-01650-0

**Published:** 2025-12-17

**Authors:** Kirtan Joshi, Kanve N. Suvilesh, Nagabhishek Sirpu Natesh, Yariswamy Manjunath, Jared Coberly, Sarah Schlink, Jeffrey R. Kunin, Randall S. Prather, Kristin Whitworth, Benjamin Nelson, Jeffrey N. Bryan, Timothy Hoffman, Mojgan Golzy, Murugesan Raju, Emma Teixeiro, Bhanu P. Telugu, Jussuf T. Kaifi, Satyanarayana Rachagani

**Affiliations:** 1 Department of Medicine/Ellis Fischel Cancer Center, University of Missouri, Columbia, MO USA; 2 Department of Surgery /Ellis Fischel Cancer Center, University of Missouri, Columbia, MO USA; 3 Roy Blunt NextGen Precision Health Institute, University of Missouri, Columbia, MO USA; 4 Harry S. Truman Memorial Veterans’ Hospital, Columbia, MO USA; 5 Department of Clinical Pathology, University of Missouri, Columbia, MO USA; 6 Office of Animal Resources, University of Missouri, Columbia, MO USA; 7 Department Of Radiology, University of Missouri, Columbia, MO USA; 8 Animal Science Research Center of the National Swine Resource and Research Center, University of Missouri, Columbia, MO USA; 9 Department of Veterinary Medicine and Surgery, University of Missouri, Columbia, MO USA; 10 Biomedical Informatics, Biostatistics and Medical Epidemiology, University of Missouri, Columbia, MO USA; 11 Institute for Data Science and Informatics, University of Missouri, Columbia, MO USA; 12 Department of Molecular Microbiology and Immunology, University of Missouri, Columbia, MO USA; 13 Alvin J. Siteman Cancer Center, Washington University, Saint Louis, MO USA

**Keywords:** Cancer models, Lung cancer

## Abstract

There remains a need for animal models with human translatability in lung cancer (LC) research. Findings in pigs can have a substantial impact owing to their similar anatomy and physiology to humans. Here we present a bronchoscopically induced LC model in Oncopigs carrying inducible *KRAS*^*G12D*^ and *TP53*^*R167H*^ mutations. A total of 12 Oncopigs underwent 29 injections via flexible bronchoscopy: 18 adenovirus–Cre recombinase gene (AdCre) inductions were performed endobronchially (*n* = 6) and transbronchially (*n* = 12), and 11 control injections were performed without AdCre. Oncopigs underwent serial chest CT with clinical follow-up for 29 weeks. Lung and organ tissues underwent histopathology, immunohistochemistry and RNA sequencing with comparative analysis to human LC data. All 18 sites of AdCre injections had lung consolidations on computed tomography imaging. Inductions led to an overall success rate of 77.8%, including both invasive cancer (61.1%) and carcinoma in situ (16.7%). Transbronchial injections led to histopathologic invasive cancer and/or carcinoma in situ in 11/12 (91.7%) and invasive cancer in 8/12 (66.6%). Endobronchial inductions led to invasive cancer in 3/6 (50%). A soft tissue metastasis was observed in one Oncopig. Immunohistochemistry confirmed the expression of Pan-Cytokeratin (Pan-CK)^+^ in epithelial cancer cells, with macrophage and T cell infiltration in the tumor microenvironment. Transcriptome comparison showed 54.3% overlap with human LC, while KRAS-mutant mouse LC had 29.88% overlap with human LC. The immunocompetent Oncopig model has a high rate of LC following bronchoscopic transbronchial induction. An overlap of the Oncopig LC transcriptome with the human LC transcriptome was noted. This pig model of LC is expected to have high clinical translatability.

## Abstract (web summary)

This study presents a bronchoscopically induced lung cancer model in Oncopigs with inducible *KRAS* and *TP53* mutations. Showing high induction rates and human-like tumoral gene expression, the model offers a translational complement to rodent systems.

## Main

Animal models, with the majority being mice^1^, are critical for advancing diagnostic and therapeutic discoveries in the care of patients with lung cancer (LC). Mouse models have substantially advanced our understanding of cancer biology and have provided foundational insights into disease mechanisms. Additionally, large animal models such as pigs offer valuable complementary insights^2–4^ as they share considerable similarities with humans in terms of lifespan, body size, anatomy, physiology, diet, metabolism, immune system characteristics and genetic features^2,3,5,6^. These attributes make pigs particularly valuable for translational cancer research aimed at more effectively bridging preclinical findings to clinical applications, as seen with pig models of cystic fibrosis^7^. Testing in pig models allows for short- and long-term trialing of human-grade novel medical/surgical devices, imaging/radiation/theranostics technologies and drugs^8^.

Oncopigs are a transgenic pig line carrying the common tumor suppressor gene *TP53*^*R167H*^ and oncogene *KRAS*^*G12D*^ mutations that are inducible in any organ of choice utilizing a *Cre/Lox* system^9–11^. Both mutations are orthologous to the mutations observed in human cancers^10–13^. While *TP53* is the most commonly mutated tumor suppressor gene in human LC^12^, *KRAS* is the most frequently mutated oncogene in non-small cell lung cancer (NSCLC) adenocarcinoma^14^. The immunocompetent Oncopig model carrying those two predominant and deterministic driver mutations of cancers offers outstanding opportunities to study LC through lung-specific, targeted induction via adenoviral-mediated Cre recombinase (AdCre) delivery^15,16^. In previous studies, Oncopig liver and pancreatic cancers were induced via organ-specific induction with AdCre, also demonstrating striking similarities with the matched human cancers^17,18^.

A recent seminal pilot study on four Oncopigs led to histopathologically confirmed LC induction at a maximum rate of 33.3% via percutaneous and 10% via endovascular injection with a surveillance period of up to 21 days post-induction^19^. Considering these relatively low LC induction rates and the substantial efforts, logistics and costs associated with large animal studies, our goal was to build on this pilot study and increase LC induction success rates by choosing a targeted, bronchoscopic induction method to develop the most efficient and reproducible Oncopig LC model. To refine the LC induction procedures, we used a higher sample size. We performed targeted AdCre injections endo- and transbronchially via minimally invasive flexible bronchoscopy. To characterize the tumor growth kinetics, we extended the surveillance period substantially to 29 weeks (performing the last computed tomography (CT) imaging at 26 weeks), while also performing autopsies at various earlier time points.

High-resolution, contrast-enhanced CT chest imaging showed consolidations at all injection sites, but none in controls (no AdCre). Upon autopsy, invasive LC and distant metastatic disease were confirmed histopathologically as Pan-CK^+^ cells, with transbronchial injections leading to the highest LC and carcinoma in situ (CIS) induction rates of >90%. LCs showed substantial immune cell infiltration in the tumor microenvironment. A comparison with orthologous human LC gene expression data from The Cancer Genome Atlas (TCGA) demonstrated much higher concordance of transgenic pig LC than *Kras*-mutant mouse LC. It is anticipated that an efficient LC Oncopig model carrying human patient-relevant mutations and showing substantial transcriptome overlap will leverage translational LC research findings for patients with LC and, ultimately, improve their outcomes.

## Results

### Bronchoscopic LC induction in Oncopigs

**Fig. 1:**
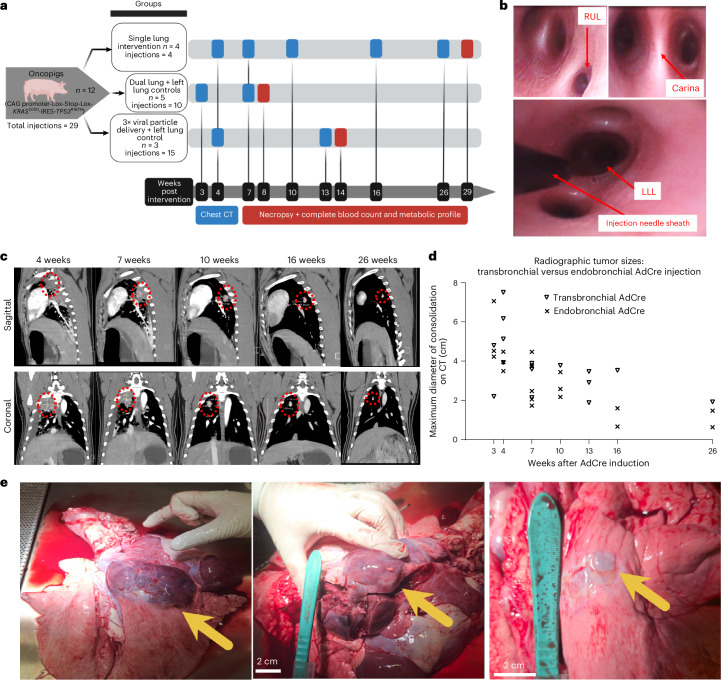
Generation of the LC oncopig. **a**, Study design: bronchoscopic inductions (*n* = 29) were performed in 12 Oncopigs separated into three groups. Clinical surveillance and follow-up imaging were performed with contrast-enhanced chest CT at the given time points. Blood analyses and necropsies were conducted at three different time points post-induction. **b**, Flexible bronchoscopy images from Oncopig airways. Left top: the right upper lobe (RUL) bronchus located proximally to the main carina. Right top: a close-up view of the main carina with bifurcation into right and left main stem bronchus. Bottom: the bronchoscopic injection needle sheath visible in the left lower lung lobe (LLL). All interventions in pigs were performed with human-grade bronchoscopy equipment. **c**, High-resolution, contrast-enhanced chest CT scans following bronchoscopic AdCre induction in Oncopigs. Representative sagittal and coronal images showing consolidation (red circle) in the lung at the AdCre injection sites at various time points of Oncopig no. 191 (group 1). Regression of the consolidations was observed over subsequent imaging, with persistence of solid lung nodules. Contralateral lung lobe injections with adjuvant agents (IL-8 and polybrene) without AdCre showed no consolidations on CT imaging. **d**, Curves showing the radiographic consolidation on CT imaging determined by maximum consolidation diameter (cm) measurements according to the AdCre injection techniques (endo- versus transbronchial between different groups). **e**, Autopsy images from Oncopigs that had undergone LC induction with bronchoscopic injection of AdCre. Left: RUL mass. Middle: the same dissected tumor. Right: RUL nodule (arrows indicate tumors).

Twelve Oncopigs (*n* = 12; 8 (66.6%) females and 4 (33.3%) males) were included for LC induction via flexible bronchoscopy (details provided in Table 1). Injections were performed in three cohorts of pigs, 9–14 weeks of age: group 1, single lung intervention (*n* = 4 Oncopigs; autopsied week 29); group 2, dual lung intervention with left lung controls (*n* = 5 Oncopigs; autopsied week 8); and group 3, multiple lung intervention, with increased adenovirus–Cre particle delivery (with 3× more quantity than in groups 1 and 2) and left lung control (*n* = 3 Oncopigs; autopsied week 14) (Fig. 1a). Slight variations in the use of adjuvants polybrene and IL-8 between groups and the increased AdCre dose in group 3 were used to adapt the experimental design according to the findings in the first cohort. These findings include histopathological lack of invasive cancer in group 1, histopathological evidence of immune cell infiltrates in the tumor microenvironment (abundance of mononuclear inflammatory cells/histiocytes, granulomata and multinucleated giant cells; and immunohistochemical confirmation of macrophages and T cells as described below) and radiographical (CT imaging) evidence for inflammatory-appearing infiltrates at the AdCre injection sites observed in groups 1 and 2. In 12 Oncopigs, a total of 29 bronchoscopic injections were performed, out of these 9 (31%) endobronchially and 20 (69%) transbronchially with a needle (Fig. 1b). A total of 18 (62.1%) LC induction attempts with AdCre were performed, while 11 (37.9%) control injections were done without AdCre, but with adjuvants polybrene (PB) ± IL-8 or phosphate-buffered saline (PBS) only. AdCre was injected endobronchially (*n* = 6/18 (33.3%)) and transbronchially (*n* = 12/18 (66.7%)) into different lung lobes (Table 1). No complications, such as endobronchial bleeding or pneumothorax, were encountered with the transbronchial needle injection.

**Table 1:** Oncopig characteristics, injection/procedure details, autopsy timing and histopathology findings

Group	Pig (no.)	Sex	Age (weeks)	Weight (kg)	Injection (number)	Injection method	Injection agents	Lung injection location		Autopsy (week)	Histopathology (invasive cancer or CIS)
								Side	Lobe		
1	200	M	9	28	1	Endobronchial	AdCre + IL-8 + PB	Right	Upper	29	No
	191	F	9	29	2	Endobronchial	AdCre + IL-8 + PB	Right	Upper		No
	192	F	9	24	3	Transbronchial	AdCre + IL-8 + PB	Right	Upper		No
	193	F	9	27	4	Endobronchial	AdCre + IL-8 + PB	Right	Upper		No
2	12-2	F	9	29	5	Transbronchial	AdCre + IL-8 + PB	Right	Upper	8	Yes
					6	Transbronchial	IL-8 + PB	Left	Lower		No
	12-5	F	9	32	7	Endobronchial	AdCre + IL-8 + PB	Right	Upper		Yes
					8	Endobronchial	IL-8 + PB	Left	Lower		No
	12-6	F	9	33	9	Endobronchial	AdCre + IL-8 + PB	Right	Upper		Yes
					10	Endobronchial	PB	Left	Lower		No
	12-8	M	9	34	11	Endobronchial	AdCre + IL-8 + PB	Left	Lower		Yes (+ soft tissue metastasis)
					12	Endobronchial	IL-8	Right	Upper		No
	12-9	M	9	32	13	Transbronchial	AdCre + IL-8 + PB	Right	Upper		Yes
					14	Transbronchial	PB	Left	Lower		No
3	54-4	F	14	33	15	Transbronchial	AdCre + IL-8	Right	Upper		
14	Yes										
					16	Transbronchial	IL-8	Right	Middle		No
					17	Transbronchial	PBS buffer only	Left	Lower		No
					18	Transbronchial	AdCre + IL-8	Left	Upper		Yes
					19	Transbronchial	AdCre only	Left	Lower		Yes
	54-8	F	14	36	20	Transbronchial	AdCre + IL-8	Right	Upper		Yes
					21	Transbronchial	AdCre + IL-8	Right	Middle		Yes
					22	Transbronchial	AdCre only	Right	Lower		Yes
					23	Transbronchial	IL-8	Left	Upper		No
					24	Transbronchial	PBS buffer only	Left	Lower		No
	54-12	M	14	34	25	Transbronchial	AdCre + IL-8	Right	Upper		Yes (CIS)
					26	Transbronchial	AdCre + IL-8	Right	Middle		Yes (CIS)
					27	Transbronchial	AdCre only	Right	Lower		Yes (CIS)
					28	Transbronchial	IL-8	Left	Upper		No
					29	Transbronchial	PBS buffer only	Left	Lower		No

F, female; M, male.

### Clinical surveillance and high-resolution chest CT imaging following induction

Oncopigs were monitored clinically and with high-resolution, contrast-enhanced chest CT imaging at several time points until autopsy (Fig. 1a). All pigs remained healthy with expected weight gains during the entire observation period. Complete blood counts (Supplementary Table 1) and basic metabolic panels (Supplementary Table 2) were measured at baseline before induction and at the time of autopsy, and they remained within normal limits (Supplementary Tables 1 and 2). All 18 (100%) AdCre injection sites led to radiographic lung consolidations at the injection site on the first contrast-enhanced chest CT (performed at 3 weeks in group 2 and at 4 weeks in groups 1 and 3) (Fig. 1c). None of the 11 control injection sites without AdCre showed any consolidations. On CT imaging, consolidations in the lung were peaking in size at the first imaging at 3 weeks (group 2) (endobronchial: mean 5.27 cm (s.d. ± 1.56), median 4.52 cm (range 4.23–7.06 cm); transbronchial: mean 3.50 cm (s.d. ± 1.84), median 3.50 cm (range 2.20–4.80 cm)), subsequently regressing while there remained persistent nodules radiographically (Fig. 1d). Of note, one Oncopig developed a pleural effusion that resolved. In some Oncopigs, a rather mild mediastinal lymphadenopathy was interpreted on CT as reactive. At the latest radiographic time point at 26 weeks, lung consolidations from endobronchial injections (mean 1.05 cm (s.d. ± 0.60), median 1.05 cm (range 0.63–1.47 cm)) were smaller than the transbronchial injection (1.92 cm (*n* = 1)) (Fig. 1d). CT imaging was performed at different, partially overlapping time points (3, 4, 7, 10, 13, 16 and 26 weeks) in the three groups. There were two time points (week 4 and week 7) where two of the groups were measured simultaneously. At week 4, group 3 (transbronchial) did show larger maximum tumor diameters compared to group 1 (endobronchial), yet not reaching the level of significance (*P* = 0.100; Mann–Whitney test) (Supplementary Fig. 1). At week 7, no significant differences were observed between the endo- versus transbronchial injections of groups 1s and 2 (*P* = 0.807; Kruskal–Wallis test). No radiographic signs of organ metastases were observed on CT imaging at any time point in the groups.

### LC induction rates are highest with transbronchial injection

At the time of autopsy (group 1, 29 weeks; group 2, 8 weeks; group 3, 14 weeks), all 18 (100%) AdCre injection sites had macroscopic masses in the lungs (Fig. 1e). There was no macroscopic evidence of metastasis in the locoregional or distant lymph nodes. All other organs inspected (including brain, liver, adrenals and kidneys) were normal, except for an incidental finding of soft tissue (subcutaneous) mass in one Oncopig (12-8 in group 2). None of the 11 control injection sites revealed macroscopic lung tumors on autopsy.

**Fig. 2:**
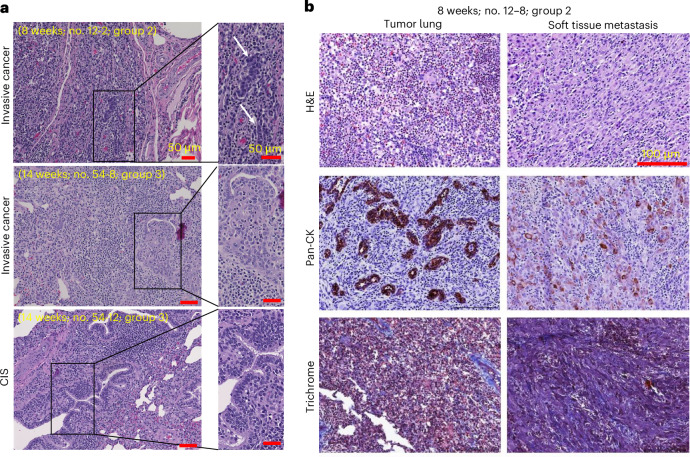
Histopathological characterization of LC oncopig tissues. **a**, H&E staining of lung tumors of three representative Oncopigs (no. 12-2 (top): group 2, autopsy at 8 weeks; no. 54-8 (middle), no. 54-12 (bottom): group 3, autopsy at 14 weeks)) showing invasive cancer cells (top and middle) and CIS (bottom). **b**, Histopathology and immunostaining of matched Oncopig (no. 12-8; group 2) LC (left) and soft tissue metastasis (right) stained with H&E (top), Pan-CK for epithelial cells (middle) and Masson’s trichrome for visualization of connective tissue/stroma (bottom).

Tumors, adjacent healthy-appearing lungs, soft tissue metastasis and multiple organ tissues were sectioned and stained. Hematoxylin & eosin (H&E) and immunohistochemistry staining were done to identify invasive, Pan-CK^+^ LC cells (Table 1). Out of the total 18 endobronchial and transbronchial AdCre injection sites, 11 (61.1%) revealed invasive cancer cells and 3 (16.7%) CIS, that is, 14/18 (77.8%) injection sites had CIS or invasive cancer (Fig. 2a and Table 1). Cancers appeared as undifferentiated NSCLC without a typical histological pattern of an adenocarcinoma or squamous cell carcinoma. In addition, no small cell carcinoma (SCLC) features were identified histopathologically.

Transbronchial injections were more efficient than endobronchial injections in LC induction and led to CIS or invasive cancer in 11/12 (91.7%), and to invasive cancer (excluding CIS) in 8/12 (66.6%). By contrast, endobronchial AdCre injections led to invasive cancer in 3/6 (50%), with no detection of CIS only. Comparing the induction of invasive cancer and CIS, transbronchial injections seemed more successful than endobronchial injections, although not reaching the level of significance (*P* = 0.083; Fisher’s exact test). No CIS/cancer was found at any of the 11 control injection sites, and no cancer cells were observed in any of the adjacent healthy lung tissues, draining hilar/mediastinal lymph nodes or other organs (Table 1). One Oncopig had a soft tissue metastasis consisting of invasive, Pan-CK^+^ cancer cells (Fig. 2b). Of note, group 3 (that received 3× higher AdCre delivery load in comparison to groups 1 and 2) showed invasive cancer at 6/9 (66.7%) of the AdCre injection sites. CIS was noted in the remaining 3/9 (33.3%) AdCre injection sites (Fig. 2a, bottom). The lower AdCre load injected in group 2 (in contrast to the highest AdCre load injected in group 3) was not associated with a lower LC induction rate. Groups 2 and 3 had equally successful induction rates. Masson’s trichrome stain showed a moderate presence of connective tissue in the primary LCs and soft tissue metastasis (Fig. 2b).

### Immune cell infiltration in the Oncopig LC tumor microenvironment

**Fig. 3:**
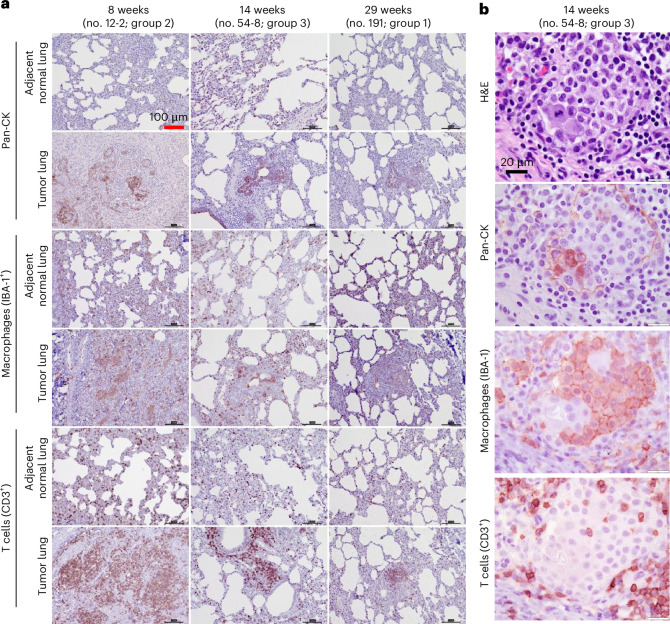
Immunostaining of oncopig tissues to check the abundance of T cell and macrophage infiltration. **a**, Representative images showing immune cell infiltration in the Oncopig LC tumor microenvironment at two different autopsy dates: at 8 weeks (Oncopig no. 12-2 (group 2)) and 14 weeks (Oncopig no. 54-8 (group 3)) post-induction. Pan-CK staining is shown. Immunohistochemistry staining for IBA-1 (macrophages) and CD3 (T cells) identified immune cells in LCs and adjacent normal lungs at 8 weeks and shows that the extent of infiltration subsequently decreased. **b**, Representative images showing T cell and macrophage infiltration around invasive cancer cells in the tumor microenvironment of an Oncopig LC (Oncopig no. 54-8 (group 3)) at higher magnification. Histopathology (H&E) staining showed the abundance of immune cells, while immunohistochemical staining identified Pan-CK^+^ epithelial cells, in addition to IBA-1^+^ macrophages and CD3^+^ T cells in the tumor microenvironment.

Substantial immune cell infiltration (including histiocytes, multinucleated giant cells and some granulomata) was observed histopathologically on H&E staining in all 18 AdCre injection sites. Immune cell-specific immunohistochemistry identified macrophages (Ionized Calcium-Binding Adapter molecule 1 (IBA-1)^+^) and T cells (CD3^+^) in the tumor microenvironment (Fig. 3). Figure 3a shows immunohistochemistry for epithelial cells (Pan-CK), macrophages (IBA-1^+^) and T cells (CD3^+^) from groups 2 and 3, with autopsy dates at 8 and 14 weeks, respectively. Although the groups were induced with slight variations, these different autopsy dates suggested initially abundant immune cell infiltration at 8 weeks following AdCre injection at the tumor sites, decreasing at week 14. Consistent with the regression of the consolidation noted on CT imaging, the overall quantity of immune cell infiltration in the tumor microenvironment decreased with a longer surveillance period.

### LC cell expression suggests cell proliferation and EMT as biomarkers of malignancy and metastatic potential

**Fig. 4:**
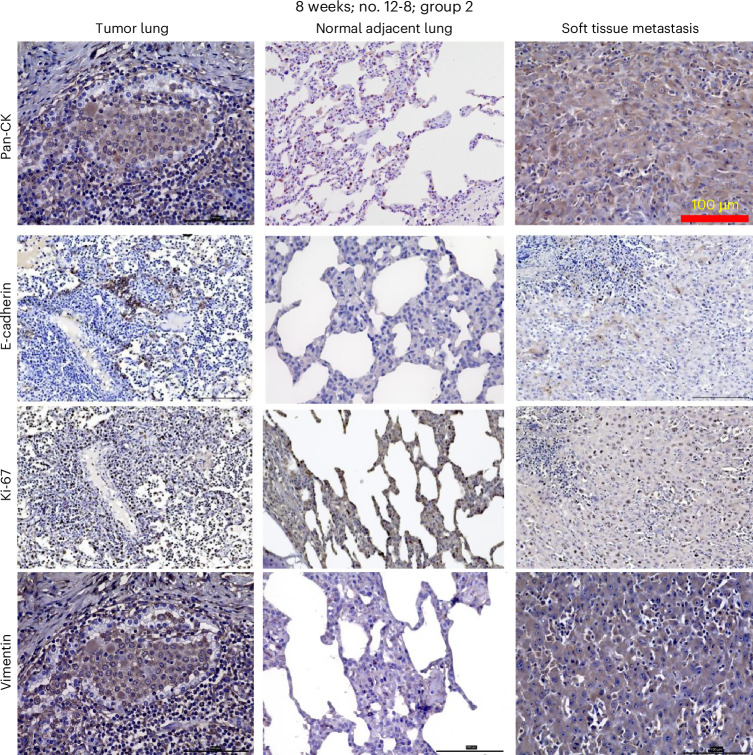
Immunostaining of oncopig tissues to check the expression of established tumor, proliferation and EMT markers. Immunostaining on LC, normal adjacent lung and soft tissue metastasis (matched) (Oncopig no. 12-8 (group 2) with endobronchial AdCre induction, autopsy after 8 weeks), showing decreased expression of E-cadherin (second row) in the metastasis. The nuclear expression of proliferation marker Ki-67 (third row) and the EMT marker vimentin (fourth row) was observed in primary LC (left), adjacent normal lung tissue (middle) and skin metastasis (right). Pan-CK expressions are also shown (top).

Immunohistochemistry for proliferation and epithelial–mesenchymal transition (EMT)-associated markers was also performed in Oncopig-derived LC tissues and matched soft tissue metastasis (Fig. 4), showing decreased expression of E-cadherin in the soft tissue metastasis compared to the primary LC. A similar expression pattern of proliferation marker Ki-67 between primary LC and the soft tissue metastasis was also noted. By contrast, higher expression of the EMT marker vimentin was observed in the soft tissue metastasis. These findings demonstrate cancer cell proliferation and metastasis-associated EMT marker expression patterns in Oncopig-derived LC tissues. KRAS mutant-specific (G12D) immunohistochemistry also confirmed expression in CIS and invasive cancer (Supplementary Fig. 2a,b). In addition, lower TP53 gene expression was found in LC tissue of the Oncopig compared to normal lung (Supplementary Fig. 2c).

### Gene expression of Oncopig LC has a substantial overlap with human LC

**Fig. 5:**
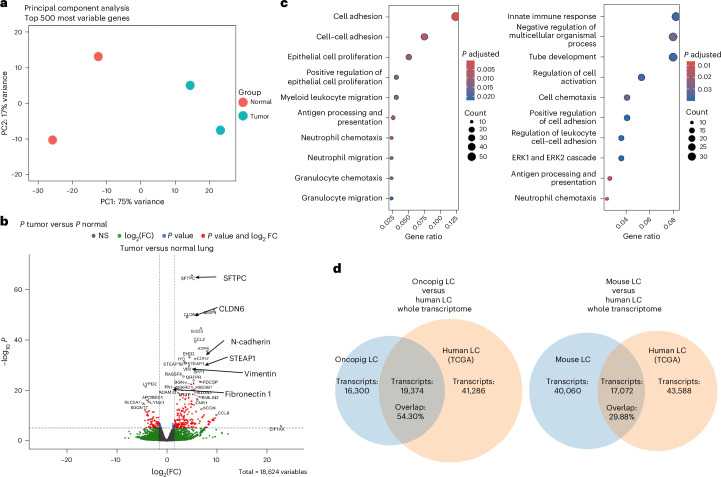
Whole-transcriptome analysis of lung tumors and healthy lung tissue from Oncopig in comparison with human LC TCGA data. **a**, Principal component analysis of Oncopig-derived LC and normal Oncopig lung showing similarities and variabilities between normal and tumor tissues (*n* = 2, respectively). **b**, Enhanced volcano plot of differential gene expression analysis between the LC tumor and normal pig lung tissues. **c**, Gene Ontology and pathway enrichment analysis of the top 50 differentially expressed genes of Oncopig LCs. **d**, Orthologous gene expression comparison with human patients with LC was performed using TCGA data (transcriptomic data from *n* = 40 patients with NSCLC (*n* = 10 per stage I–IV, randomly selected)), demonstrating 54.3% of the Oncopig LC transcripts overlapping with human LC transcripts, in contrast to less (29.88%) overlap with mouse *KRAS*-mutant LC.

Gene expression analysis of Oncopig-derived LC tumor tissues and normal Oncopig lung tissue (*n* = 2, respectively) was performed by whole-transcriptome sequencing to identify the molecular expression landscape and allow comparison with data from human patients with LC (Fig. 5). Principal component analysis revealed distinct changes in the transcriptome between Oncopig tumor samples versus normal pig lung tissues (Fig. 5a). Differential gene expression analysis showed overexpression of classic epithelial genes (for example, *SFTPC* and *STEAP1*), genes associated with cancer (for example, *CLDN6*) and regulators of EMT (for example, *VIM*, *FN 1* and *CDH2*) in Oncopig LC versus normal pig lung tissues (Fig. 5b). These gene expression changes demonstrate cancer-specific transcriptome changes in Oncopig LC. Further, Gene Ontology enrichment analysis of the top 50 differentially expressed genes between LC and normal tissues showed cell adhesion and migration pathway enrichment in tumor tissues over normal lung tissues (Fig. 5c).

For comparative analysis of Oncopig LC with human LC gene expression data, orthologous gene expression analysis was performed using data from TCGA (n = 40 patients with NSCLC), randomly selected to avoid sampling bias (*n* = 10 per stage I–IV). This transcriptome comparison of differentially expressed genes between pig and human LC revealed 54.3% overlap of Oncopig LC with human LC transcriptome (Fig. 5d, left). A similar orthologous analysis revealed substantially less (29.88%) overlap between KRAS-mutant mouse LC and human LC (Fig. 5d, right).

## Discussion

Scientific findings in pigs are highly translatable to human patients^6,20,21^ owing to similarities in anatomy, physiology and metabolism^2,3,5,7,22,23^. Pigs also allow the use of human-grade instrumentation, which is advantageous for studying human diagnostic and therapeutic modalities, including medical devices (for example, ablation technologies), imaging/theranostics, radiation and surgical techniques^21^. We expanded the technical feasibility of a transgenic pig model for LC in a larger pig cohort with a longer surveillance period to develop a robust preclinical LC model. Transbronchial injection led to a successful tumor induction rate of invasive cancer and CIS (>90%), substantially higher than endobronchial injection in our cohort and endovascular/percutaneous injection in a recent pilot study^19^. No procedure-associated complications were encountered with the transbronchial needle injections, which can be performed with minor operator training in a minimally invasive fashion using a flexible bronchoscope, without the need for radiographic image guidance with radiation exposure. In addition, transbronchial injections allowed for an AdCre depot injection with a presumably higher local concentration into a reproducible lung target area, rather than a more diffuse endobronchial or endovascular injection that may not result in sufficient AdCre concentrations for tumor induction. A percutaneous lung intervention, such as a percutaneous needle injection, has a significant risk of a pneumothorax owing to violation of the visceral pleura, which can also be avoided with a more central transbronchial needle injection of AdCre that does not violate the visceral pleura. Histopathologically, the LCs showed substantial tumor microenvironment immune cell infiltration, opening avenues to study resistance mechanisms toward immunotherapy in LC. Oncopig LC cells also expressed cancer-associated biomarkers, with gene expression matching that of human NSCLC at a high rate of >54%.

Most cancer treatment effects that have been investigated in a variety of mouse models^24–32^ have subsequently not been reproducible in human clinical trials^20^. More recently, patient tissue-derived tumor organoids have evolved as promising drug testing platforms for personalized medicine^33–35^, but all human-derived models are limited by a lack of repeated tissue availability, loss of local tumor microenvironment and inability to represent the systemic immune responses^29^. So far, pig cancer models have also faced challenges beyond complex maintenance and high costs. For instance, human cancer cell lines xenografted into pigs^36^ require the use of immunodeficient animals that are unsuitable for immunotherapy study^37^. Other pigs have been genetically engineered to express a gene of interest with a reporter, but the absence of mutations in oncogenic driver genes (similar to humans) limited the clinical applicability of the model^38^. In addition to being immunocompetent, the Oncopig^9^ is transformative as it carries the most common mutant tumor suppressor (*TP53*^*R167H*^) and a commonly found (20–50%, depending on the cancer type^12^) oncogenic mutation (*KRAS*^*G12D*^) orthologous to human cancer mutations^10,11^ that can be induced orthotopically in the lung.

In a pilot study of four Oncopigs, LC was induced differently by AdCre delivery with endovascular and percutaneous injection^19^. The surveillance was 21 days and neoplastic lung nodules developed in 10% of endovascular and in 33% of percutaneous injections, respectively^19^. Our study expanded the cohort to 12 Oncopigs with longer surveillance and ultimately accomplished higher (>90%) LC induction via transbronchial needle injections. Radiographically, tumor sizes were the largest at 3–4 weeks post-induction, offering a large animal LC model to perform short-term and long-term studies. We confirmed the presence of epithelial cancer cells that expressed EMT and other cancer biomarkers, whereas the tumor microenvironment contained abundant macrophages and T cells^19^. Of note, all CIS were observed following transbronchial injections with a higher AdCre dosage in group 3, after a follow-up of 14 weeks. In one Oncopig, a soft tissue metastasis was identified incidentally on autopsy with histopathological confirmation. This soft tissue metastasis was the only extrapulmonary site of disease identified and may represent either a true hematogenous metastasis or an accidental event, such as inadvertent intravascular AdCre delivery during transbronchial injection. As described in the previous pilot study^19^, we also found desmoplastic stroma and undifferentiated rather non-small cell cancers, without typical characteristics of adenoid or squamous cell subtypes of NSCLC and no features of SCLC.

The inflammatory reaction with macrophages and T cells infiltrating the LC tumor microenvironment could benefit future studies on immunotherapy responses. Of note, the Oncopig was used for pancreatic cancer induction with a success rate of 71%, and the investigators also reported severe inflammation/pancreatitis^39^. Oncopigs with pancreatic injections of adjuvant IL-8 without AdCre and wild-type pigs with AdCre injection did not show notable inflammation^39^, indicating that the tumor-associated inflammation may be secondary to an immune response to an acute load of tumor-associated neoantigens^19,39^. Our study did not observe any inflammatory reactions at the control injection sites with adjuvants without AdCre. An immune response from AdCre injection within the Oncopig skeletal muscle has been characterized as an intratumoral infiltration of cytotoxic T cells with enrichment of regulatory (FOXP3^+^) T cells, with increased expression of immune checkpoint inhibitor targets indoleamine 2,3-dioxygenase 1 (IDO1), cytotoxic T-lymphocyte-associated protein 4 (CTLA4) and programmed death-ligand 1 (PDL1)^40^. These findings were interpreted as a beneficial antitumor immune response and support the hypothesis that the Oncopig may serve as a valuable model to study tumor-directed cytotoxicity^40^. Compared to rodents, the porcine immune system is much more similar to humans, again suggesting higher translatability^41^.

Upon further molecular characterization of cancer hallmark biomarkers, we found a downregulation of the expression of cell–cell adherens junction transmembrane protein E-cadherin and upregulation of EMT marker vimentin as indicators of metastatic capacity. These results are comparable to the findings observed in the Oncopig pancreatic cancer model^39^. Increased cell proliferation was also observed. Compared to untransfected Oncopig lung tissue, Oncopig LC tissue showed higher differential expression of genes related to cell adhesion and proliferation and the EMT. Importantly, the transcriptome comparison of Oncopig LC (*n* = 4) with human TCGA NSCLC patient data (*n* = 40) showed a striking overlap of 54.30%. We also performed a similar comparison^42^ using data from LCs generated in KRAS-mutant mice^43^, which revealed substantially less overlap (29.88%) of orthologous genes between mouse and human LC. Our findings in LC are consistent with previous cross-species transcriptome analyses that revealed a low proportion of ortholog transcripts (<20%) between mice and humans^44^, further supporting the high value of pigs for translational research.

There are several limitations to consider in our pig study, which was associated with substantially higher costs and logistical efforts compared to rodent studies. Foremost, we did not achieve a 100% induction success rate. However, we accomplished higher cancer induction efficiency than in any other Oncopig cancer study, with the transbronchial injections leading to >90% of at least CIS. Owing to our low sample size of 12 Oncopigs, we also allowed for some interpretation bias as we administered different dosages of AdCre and modified adjuvant compositions to enhance the chance of LC induction. Furthermore, there is limited availability of anti-porcine antibodies, restricting extensive molecular and pathological characterization for subtyping of Oncopig LCs. Finally, a specific consideration is that the current Oncopig line carries the quite prevalent *KRAS*^*G12D*^ mutation, which is more common in gastrointestinal cancers^45^. The glycine-to-cysteine mutation in position 12 (*KRAS*^*G12C*^) is the most common *KRAS* mutation in LC (>15%)^14^, with available FDA-approved drugs (for example, sotorasib)^46^. An Oncopig line carrying *KRAS*^*G12*C^ would be most appropriate for studying KRAS-associated drug resistance in LC.

## Conclusion

The transgenic, immunocompetent and orthotopic LC Oncopig model is expected to have a clinical impact for human LC care. Preclinical testing in an Oncopig LC model (including bronchoscopic interventions, ablations, medical devices, radiation approaches, theranostics and targeted and immunotherapies)^47^ could be impactful, as various modalities are challenging or impossible to test in smaller animals. A highly translatable pig LC model is anticipated to lead to novel findings for precision oncology to improve the outcomes of patients with LC.

## Methods

### Animals

This single-institution prospective study was approved by the Institutional Animal Care and Use Committee at the University of Missouri (protocol no. 18120). All animal housing facilities are accredited by the American Association of the Accreditation of Laboratory Animal Care and are in compliance with and regulated by the USDA (43R0048) and the Office of Laboratory Animal Welfare (D16-00249). Generation of the Oncopig line is described in Schook et al.^9^. Male and female 9–14-week-old Minnesota mini Oncopigs (LSL-KRASG12D-IRES-TP53R167H) were acquired from the National Swine Research and Resource Center (NSRRC) and transported to the Animal Sciences Research Center at the University of Missouri, Columbia. The animals were allowed to acclimate to the facility for 3 days before the bronchoscopic induction interventions. Within 3 days after the bronchoscopic injections, the animals were transported to the NextGen Vivarium at the University of Missouri, where all subsequent procedures (including CT imaging) were performed and where pigs were kept until autopsy. Pigs were under the constant care of clinical veterinarians with the support of specialized animal care staff providing husbandry and care support for the animals and a host of residents providing 7-day per week veterinary care. Pigs were housed individually or in small groups in pens. The floor area (square foot) per animal was weight adjusted, ensuring that individually housed pigs had visual, olfactory and auditory contact with other pigs in different pens to prevent social deprivation. Drinking water was constantly available, and food (Purina Probuild Team Lean,Purina Animal Nutrition) was provided twice daily based on the growth requirements of pigs. Pigs were monitored daily and weights were measured weekly. In all pig experiments, the maximal tumor size or burden was not exceeded as per the approved protocol and guidelines of the institutional animal care and use committee protocol.

### Endo- and transbronchial injections of AdCre

Ad5CMVCre-eGFP (high titer) virus was obtained from the University of Iowa Viral Vector Core. A 1 × 10^11^ plaque-forming unit (p.f.u.)/ml concentration was used for groups 1 and 2. A volume of 150 µl of AdCre (1 × 10^11^ p.f.u./ml) ± adjuvants porcine IL-8 (5 ng/ml) (NBP2-35234; Novus Biological) and ± adjuvants PB (1:100) were injected. In group 3, we chose to give a 3× higher dose (3 × 10^11^ p.f.u./ml) of AdCre, as had been suggested for the development of a pancreatic cancer transgenic Oncopig model^39^. The rationale for using cytokine IL-8 with AdCre is that it mobilizes the adenovirus receptor to the luminal membrane of epithelial cells, thereby enhancing viral entry^39,48,49^. PB (hexadimethrine bromide) was used to increase binding between the pseudoviral capsid and the cellular membrane to enhance transduction efficiency^50^.

Anesthesia and procedures were performed or supervised by veterinary physicians with expertise in clinical veterinary care, a general thoracic surgeon trained in airway management and interventional bronchoscopy, and ancillary veterinary staff trained in general pig care. Pigs were anesthetized using ketamine and xylazine. During interventions, the pigs were placed in a laterally recumbent position and maintained under general anesthesia using inhalation isoflurane (2 l/min) with either a nose cone or a laryngeal mask. Pigs were anesthetized for a relatively short time during the bronchoscopic inoculation and the CT scans. The average time under anesthesia was approximately 15 min or shorter.

Bronchoscopic injections were performed via laryngeal mask using an Ambu aScope 4 Bronchoscope (regular, 5.0/2.2) inserted transorally. An Interject Injection Therapy needle catheter (25-gauge, 6 mm needle) was used to inject transbronchially. Injections via flexible bronchoscopy were done using two techniques: (1) endobronchial or (2) transbronchial needle injection into different lung lobes. As controls, adjuvants (PB ± IL-8) or vehicle (PBS) alone without AdCre were injected into the contralateral lung lobes in the same pig. The bronchoscope was passed through a laryngeal mask and navigated to the target lobe (Table 1). The inoculation mixtures were injected endobronchially via the bronchoscopy channel or transbronchially using the needle (Fig. 1). For transbronchial injections, the injection needle catheter dead space was flushed with 200 µl PBS to ensure that all AdCre was inoculated.

### CT imaging

Oncopigs were clinically monitored daily for pain and distress, including coughing, tachypnea, lethargy or weight loss. If excessive coughing was witnessed in the pigs, 3.75 mg/kg of carprofen was administered daily for 5 days. For contrast-enhanced CT (CT scanner: Definition Edge 128 slice; Siemens), 2 ml/kg Omnipaque (350 mg/ml) was injected intravenously with a 15 ml saline flush. The injection rate was 2 ml/s with a 40-s delay between initiating the contrast injection and the scan. The CT scans were acquired at 120 kV and a slice of 0.5 mm with additional orthogonal reconstructions at a thickness of 2.0 mm, and the CT scanner used achieves a temporal resolution of 142 ms. The reconstructed field of view was 300 mm with a 512 × 512 matrix, resulting in a 0.585 mm/pixel size. All CT images were stored in a DICOM medical image management system (Ambra) and interpreted by a board-certified radiologist specialized in thoracic imaging (J.R.K.).

### Blood drawings, autopsy, pathological analysis and sequencing of tissues

At the time of autopsy, a minimum of 10 ml of blood was collected via a jugular vein phlebotomy and collected in K2–EDTA and heparin tubes (BD Vacutainer). Complete blood counts and basic metabolic panels were determined. Animals were euthanized using an intravenous Euthasol injection (1 ml/4.5 kg; Virbac). Following euthanasia, an autopsy with gross examination of all major organs, including thoracic and abdominal lymph nodes, was performed under the supervision of a veterinary pathologist. Tissues were fresh frozen or formalin-fixed for histopathology. Following processing in alcohol and saline, tissues were embedded in paraffin and sectioned at 5 µm thickness. Slides were stained for H&E and immunohistochemistry was performed with antibodies for Pan-CK (MNF116, mouse; Agilent), IBA-1 (ab5076, goat; Abcam) and CD3 (ab135372, rabbit; Abcam). Additional immunostaining was performed using E-cadherin (3195S, rabbit; CST), Ki-67 (9449S, mouse; CST), vimentin (V6630, mouse; Sigma) and K-Ras (G12D Mutant; rabbit; Invitrogen). Chromogen was 3,30-diaminobenzidine tetrachloride (DAB) and the counterstain was hematoxylin. Masson’s trichrome staining was performed to visualize collagen in tissues. All immunohistochemical staining was validated using appropriate swine control tissues as negative controls. Slides were then evaluated and scored blinded by a board-certified pathologist. Expression quantification analysis was performed with ImageJ^51^. Procedures for the RNA-sequencing of pig tissues are described in Supplementary Methods. All the reagents and antibodies used in the study are listed in Supplementary Table 3.

### Statistical analysis

Statistical differences between more than two groups were calculated via contingency table analysis with Fisher’s exact test. Nonparametric analyses were performed with the Mann–Whitney and Kruskal–Wallis tests. All standard statistical analyses were done using Prism v8.00 (GraphPad). Bioinformatic analyses of sequencing data and cross-species orthologous gene expression analyses are outlined in Supplementary Methods. A *P* value of <0.05 was considered significant.

### Reporting summary

Further information on research design is available in the Nature Portfolio Reporting Summary linked to this article.

## Online content

Any methods, additional references, Nature Portfolio reporting summaries, source data, extended data, supplementary information, acknowledgements, peer review information; details of author contributions and competing interests; and statements of data and code availability are available at 10.1038/s41684-025-01650-0.

## Supplementary information

Supplementary Information Supplementary Methods, Tables 1–3 and Figs. 1 and 2.

Reporting Summary

## Data Availability

All data generated or analyzed during this study, if not included in this article and its Supplementary Information files, are available from the corresponding authors on request.
