# Supplementary material for: Characterization of a bronchoscopically induced transgenic lung cancer pig model for human translatability
Source: Lab Anim (NY). 2025 Dec 17;55(1):12–20. doi: 10.1038/s41684-025-01650-0 (PMC12738287; doi:10.1038/s41684-025-01650-0)
Supplement: Supplementary file 1 — Supplementary Methods, Tables 1–3 and Figs. 1 and 2. [file 41684_2025_1650_MOESM1_ESM.pdf]

lab animal

Supplementary information

https://doi.org/10.1038/s41684-025-01650-0

Characterization of a bronchoscopically induced transgenic lung cancer pig model for human translatability

In the format provided by the authors and unedited

SUPPLEMENTARY METHODS

Whole transcriptome sequencing and bioinformatic analysis

For whole transcriptome analysis of Oncopig-derived LC tumor (n=2) and normal lung tissues (n=2), RNA extraction was done with cellular lysis using MagMax mirVana Total RNA Isolation kit (A27828; Thermo Fisher), following the manufacturer's instructions. The RNA samples were subjected to whole transcriptome analysis (RNA sequencing) at ArrayStar Inc. (Rockville, MD). Assessment of RNA quality was accomplished using NanoDrop ND-1000 instrument. ~1-2 μg total RNA was used to prepare the sequencing library. Briefly, total RNA was enriched by oligo (dT) magnetic beads (rRNA removed), followed by RNA-seq library preparation using KAPA Stranded RNA-Seq Library Prep Kit (Illumina). The completed libraries were qualified with an Agilent 2100 Bioanalyzer and quantified by the absolute quantification qPCR method. To sequence the libraries on the Illumina NovaSeq 6000 instrument, the barcoded libraries were mixed, denatured to single-stranded DNA in NaOH, captured on Illumina flow cell, amplified in situ, and subsequently sequenced for 150 cycles for both ends on Illumina NovaSeq 6000 instrument.

Pre-processing and normalization: The fastq reads obtained were preprocessed to remove low-quality reads and adapters using fastp (v0.02.0). Quality control was performed using FastQC (v0.11.9). The clean reads were aligned to the Pig reference genome (Sscrofa11.1) using STAR (v2.7.10) with default parameters. Gene expression levels were quantified, and the resulting count data were normalized for differences in library size by estimating size factors.

Principal Component Analysis (PCA): PCA was conducted to examine overall similarities and variations between experimental groups. The PCA plot was generated using the plot PCA function by plotting the first two principal components on the x and y axes. The top 500 most variable genes were used for the PCA plot.

Differential Gene Expression Analysis: DESeq2 R package (v1.42.0) was used to find differentially expressed genes (DEGs). To estimate mean expression levels between normal and tumor samples, DESeq2 with a negative binomial generalized linear model and Wald test was used to evaluate the significance of log2 fold changes. Multiple testing corrections for controlling the false discovery rate (FDR) were applied using the Benjamini-Hochberg procedure. Genes were identified as differentially expressed based on adjusted p-values < 0.05 and log2 fold changes ± 1.5. A volcano plot was generated to visualize the relationship between fold change and the statistical significance of DEGs. The enhanced Volcano function from the R package (v1.20.0) generated the plot, highlighting significant DEGs with an adjusted p-value of less than 0.05.

Gene Ontology (GO) Enrichment Analysis: GO enrichment analysis was performed to identify significantly overrepresented genes and understand the enriched biological processes. The analysis involved calculating GO terms' expected and observed frequencies and applying statistical tests (Fisher's exact test) to determine significant overrepresentation. Results were visualized as dot plots and cluster plots using the clusterProfiler R package.

Human and OncoPig Cross-Species Genome Comparison: A comprehensive comparative transcriptomic analysis was conducted to identify and compare gene expression patterns between OncoPig and human tumors. Human NSCLC transcriptome RNA-seq count data and clinical pathological information, including stage, were acquired from The Cancer Genome Atlas (TCGA) database. The data were downloaded in raw count format. Initial preprocessing involved assessing data quality, filtering out low-quality reads, and ensuring data integrity. The raw counts were further processed using the DESeq2 R package (v1.42.0) to normalize the data and mitigate variations in sequencing depth and RNA composition among samples. Orthologous gene mapping between OncoPig and human genomes was performed using orthologous gene mapping, ensuring accurate identification of corresponding genes across species. Following this, gene expression data from both species were integrated and normalized to account for differences in sequencing depth and RNA composition using the DESeq2 framework. The normalized datasets enabled a robust comparative analysis to pinpoint conserved and divergent gene expression features relevant to lung cancer. Similar analysis was performed using publicly available KRAS-mutant mouse lung cancer datasets [44] to identify the overlap of differentially expressed genes between mouse and human lung cancer. Briefly, KRAS-mutant mouse lung tumor (n=3) and normal lung (n=3) RNA-seq FastQ files were downloaded from NCBI gene expression omnibus using BioProjects PRJNA1033182 and PRJNA1090424, respectively. FastQ reads were preprocessed, and the resulting count data were used as described above to identify orthologous gene expression overlap between mouse and human lung cancer differentially expressed genes.

Supplementary Table 1: Complete Blood Count (CBC) of OncoPigs at baseline and autopsies

**Time Point: Baseline**		**Group 1**				**Group 2**					**Group 3**		
Component	Reference Interval	Pig # 200	Pig # 191	Pig # 192	Pig # 193	Pig # 12-2	Pig # 12-5	Pig # 12-6	Pig # 12-8	Pig # 12-9	Pig # 54-4	Pig # 54-8	Pig # 54-12
WBC x10^3/uL	11.30 - 22.80	8.95 **L**	12.38	N/A	11.6	15.23	12.98	15	10.8	10.41 **L**	17.4	15.04	17.37
RBC x10^6/uL	5.90 - 8.80	6.35	5.96	N/A	5.96	7.49	6.95	6.26	6.74	6.41	6.11	6.44	6.61
HGB g/dL	10.1 - 15.1	12.8	10.8	N/A	10.6	13.4	12.6	11.3	12.5	10.7	12.5	11.8	12.2
HCT %	31.1 - 45.9	37.8	32.1	N/A	31.6	41.1	38.7	33.2	38.1	33.6	36.1	32.4	35.5
MCV fL	44.2 - 60.9	59.5	53.8	N/A	53	54.8	55.7	53	56.6	52.4	59.1	50.3	53.7
MCH pg	14.4 - 20.1	20.2 **H**	18.1	N/A	17.8	17.8	18.1	18	18.5	16.8	20.5 **H**	18.4	18.5
MCHC g/dL	30.6 - 34.9	33.9	33.7	N/A	33.7	32.6	32.5	34	32.7	32	34.7	36.5 **H**	34.4
RDW %	14.8 - 19.8	17.2	17.4	N/A	17.7	19.5	16.8	19.4	22.2 **H**	21.5 **H**	15.1	16.5	16
Abs Reticulocyte x10^3 /uL	No Ref Interval	316	264	N/A	178	317	204	214	239	160	73	61	102
Platelet Count x10^3/uL	138 - 468	467	520 **H**	N/A	616 **H**	437	530 **H**	401	445	612 **H**	18 **L**	75 **L**	152
MPV fL	No Ref Interval	8.3	8.1	N/A	8.2	9.2	9	10	9.4	8.4	18.2	11.3	12.7
PCT %	No Ref Interval	0.39	0.42	N/A	0.5	0.4	0.48	0.4	0.42	0.51	0.03	0.08	0.19
**Time Point: Autopsy**		**Group 1**				**Group 2**					**Group 3**		
Component	Reference Interval	Pig # 200	Pig # 191	Pig # 192	Pig # 193	Pig # 12-2	Pig # 12-5	Pig # 12-6	Pig # 12-8	Pig # 12-9	Pig # 54-4	Pig # 54-8	Pig # 54-12
WBC x10^3/uL	11.30 - 22.80	N/A	N/A	9.41 **L**	8.83 **L**	13.75	14.8	16.03	12.36	12.17	9.62 **L**	9.56 **L**	14.72
RBC x10^6/uL	5.90 - 8.80	N/A	N/A	5.3 **L**	5.81 **L**	7.21	6.88	7.03	6.51	7.01	5.61 **L**	7.43	6.87
HGB g/dL	10.1 - 15.1	N/A	N/A	10.2	11.3	12.7	12.4	12.1	12.5	12.1	11.1	12	12.2
HCT %	31.1 - 45.9	N/A	N/A	28.8 **L**	30.1 **L**	37.3	36.7	36	35.9	35.8	32.8	35.8	36.8
MCV fL	44.2 - 60.9	N/A	N/A	54.3	51.7	51.8	53.3	51.3	55.2	51	58.5	48.1	53.6
MCH pg	14.4 - 20.1	N/A	N/A	19.3	19.4	17.6	18	17.3	19.2	17.2	19.9	16.1	17.7
MCHC g/dL	30.6 - 34.9	N/A	N/A	35.6 **H**	37.6 **H**	34.1	33.8	33.7	34.7	33.7	34	33.5	33.1
RDW %	14.8 - 19.8	N/A	N/A	16.2	17.3	17.2	15.7	16.8	18.7	18.1	15	16.1	17.8
Abs Reticulocyte x10^3 /uL	No Ref Interval	N/A	N/A	29	24	41	31	33	32	34	52	52	157
Platelet Count x10^3/uL	138 - 468	N/A	N/A	187	363	344	397	418	327	480 **H**	268	332	380
MPV fL	No Ref Interval	N/A	N/A	9.5	13.7	9	9.1	9.6	8.9	8.8	10.5	9.4	10
PCT %	No Ref Interval	N/A	N/A	0.18	0.5	0.31	0.36	0.4	0.29	0.42	0.28	0.31	0.38

Abbreviations: **H**-High, **L**-Low, N/A- Not available

Supplementary Table 2: Basic Metabolic Panels (BMP) of Oncopigs at baseline and autopsies

**Time Point: Baseline**		**Group 1**				**Group 2**					**Group 3**		
Component	Reference Interval	Pig # 200	Pig # 191	Pig # 192	Pig # 193	Pig # 12-2	Pig # 12-5	Pig # 12-6	Pig # 12-8	Pig # 12-9	Pig # 54-4	Pig # 54-8	Pig # 54-12
Glucose mg/dL	No Ref Interval	112	81	N/A	78	61	89	71	60	64	137	108	115
Urea Nitrogen mg/dL	No Ref Interval	18	17	N/A	15	18	15	16	14	12	9	9	8
Creatinine mg/dL	No Ref Interval	0.9	0.7	N/A	0.8	0.8	0.6	0.7	0.6	0.7	0.7	0.6	0.8
Sodium mEq/L	No Ref Interval	145	142	N/A	142	140	139	140	140	140	142	141	142
Potassium mEq/L	No Ref Interval	3.8	4.1	N/A	3.9	4.7	4.1	4	4.3	4.1	5	4.9	4.5
Chloride mEq/L	No Ref Interval	100	98	N/A	99	98	98	99	100	100	99	100	101
Bicarbonate mEq/L	No Ref Interval	15	25	N/A	25	27	32	31	28	27	32	33	30
Anion Gap mEq/L	No Ref Interval	34	23	N/A	22	20	13	14	16	17	16	13	16
Albumin g/dL	No Ref Interval	4.4	3.5	N/A	3.6	3.8	3.6	3.9	3.9	3.5	3.4	3.3	3.5
Total Protein g/dL	No Ref Interval	6.7	6	N/A	6.2	5.7	5.6	5.8	6.1	5.6	6.2	5.9	5
Globulin g/dL	No Ref Interval	2.3	2.5	N/A	2.6	1.9	2	1.9	2.2	2.1	2.8	2.6	1.5
Calcium mg/dL	No Ref Interval	11.6	11.3	N/A	10.3	10.7	11.1	11.3	10.6	10.6	10.6	10.2	10.4
Phosphorus mg/dL	No Ref Interval	11.4	12.3	N/A	10.8	11.2	10.9	11	10.9	10.9	9.2	9	10.1
Magnesium mg/dL	No Ref Interval	2.3	2.1	N/A	2.1	2.5	2.2	2.3	2.3	2.1	2	1.9	1.6
Total Bilirubin mg/dL	No Ref Interval	0.5	0.4	N/A	0.6	0.6	0.4	0.4	0.6	0.4	0.1	0.1	0.1
Direct Bilirubin mg/dL	No Ref Interval	0.2	0.1	N/A	0.2	0.2	0.1	0.1	0.2	0.1	0	0	0
AST U/L	No Ref Interval	36	35	N/A	49	36	38	37	36	34	22	15	18
GGT U/L	No Ref Interval	38	60	N/A	35	79	45	39	44	44	33	31	29
GLDH U/L	No Ref Interval	0	0	N/A	0	1	1	2	1	1	0	1	1
CK U/L	No Ref Interval	3986	904	N/A	5098	1014	1553	684	1600	1386	530	1481	1698
**Time Point: Autopsy**		**Group 1**				**Group 2**					**Group 3**		
Component	Reference Interval	Pig # 200	Pig # 191	Pig # 192	Pig # 193	Pig # 12-2	Pig # 12-5	Pig # 12-6	Pig # 12-8	Pig # 12-9	Pig # 54-4	Pig # 54-8	Pig # 54-12
Glucose mg/dL	No Ref Interval	102	95	72	102	128	105	107	101	96	90	105	82
Urea Nitrogen mg/dL	No Ref Interval	7	6	6	7	9	6	7	9	7	6	4	9
Creatinine mg/dL	No Ref Interval	0.7	0.7	0.8	0.8	0.9	0.8	1	0.9	1	1.1	0.9	1.3
Sodium mEq/L	No Ref Interval	123	137	136	130	137	137	138	137	138	131	125	139
Potassium mEq/L	No Ref Interval	4.2	4.3	4.1	3.8	3.7	4	4	3.9	3.8	4.9	4.9	4.3
Chloride mEq/L	No Ref Interval	87	98	98	91	97	97	99	98	101	94	90	100
Bicarbonate mEq/L	No Ref Interval	29	31	30	30	34	32	34	32	32	31	29	31
Anion Gap mEq/L	No Ref Interval	11	12	12	13	10	12	9	11	9	11	11	12
Albumin g/dL	No Ref Interval	3.4	3	2.8	2.9	3.7	3.4	4	3.9	3.8	3.6	3.6	3.7
Total Protein g/dL	No Ref Interval	5.1	5.7	5.4	5.3	5.8	5.7	6.4	6.4	6.2	6.5	6.3	7.1
Globulin g/dL	No Ref Interval	1.7	2.7	2.6	2.4	2.1	2.3	2.4	2.5	2.4	2.9	2.7	3.4

Calcium mg/dL	No Ref Interval	9.6	9.6	9.7	9.4	10.3	9.8	10.9	10.8	10.1	10.1	9.4	9.8
Phosphorus mg/dL	No Ref Interval	7.1	8	8.1	7.7	8.7	9.1	8.4	8.9	8.3	7.5	6.9	8.2
Magnesium mg/dL	No Ref Interval	1.7	2.1	1.8	2.1	1.9	1.8	2	1.8	1.7	1.8	1.5	1.8
Total Bilirubin mg/dL	No Ref Interval	0.3	0.1	0.1	0.2	0.1	0.1	0.1	0.1	0.1	0.2	0.2	0.1
Direct Bilirubin mg/dL	No Ref Interval	0.1	0	0	0.1	0.1	0	0	0	0	0	0	0
AST U/L	No Ref Interval	25	47	24	30	16	47	39	108	17	27	13	17
GGT U/L	No Ref Interval	28	38	41	31	66	68	59	52	57	41	33	33
GLDH U/L	No Ref Interval	0	1	1	1	1	1	1	1	1	1	0	1
CK U/L	No Ref Interval	1020	5245	1679	1230	277	3134	1776	7482	358	1096	255	737

Abbreviations: N/A- Not available

Supplementary Table 3: List of reagents and antibodies used in the study

**Product**	**Catalogue #**	**Company**
Ad5CMVCre-eGFP	1174	UI Viral Vector Core
Porcine IL-8	NBP2-35234	Novus Biological
Polybrene	TR-1003	Sigma-Aldrich
Phosphate-buffered saline	14190144	ThermoFisher Scientific
Pan-Cytokeratin	MNF116	Agilent
Ionized Calcium-Binding Adaptor molecule 1 (IBA-1)	ab5076	Abcam
CD3	ab135372	Abcam
E-cadherin	3195S	Cell Signaling Technology
Ki-67	9449S	Cell Signaling Technology
Vimentin	V6630	Sigma-Aldrich
MagMax mirVana Total RNA Isolation kit	A27828	ThermoFisher Scientific
K-Ras (G12D Mutant)	HL2630	Invitrogen

SUPPLEMENTARY FIGURE 1

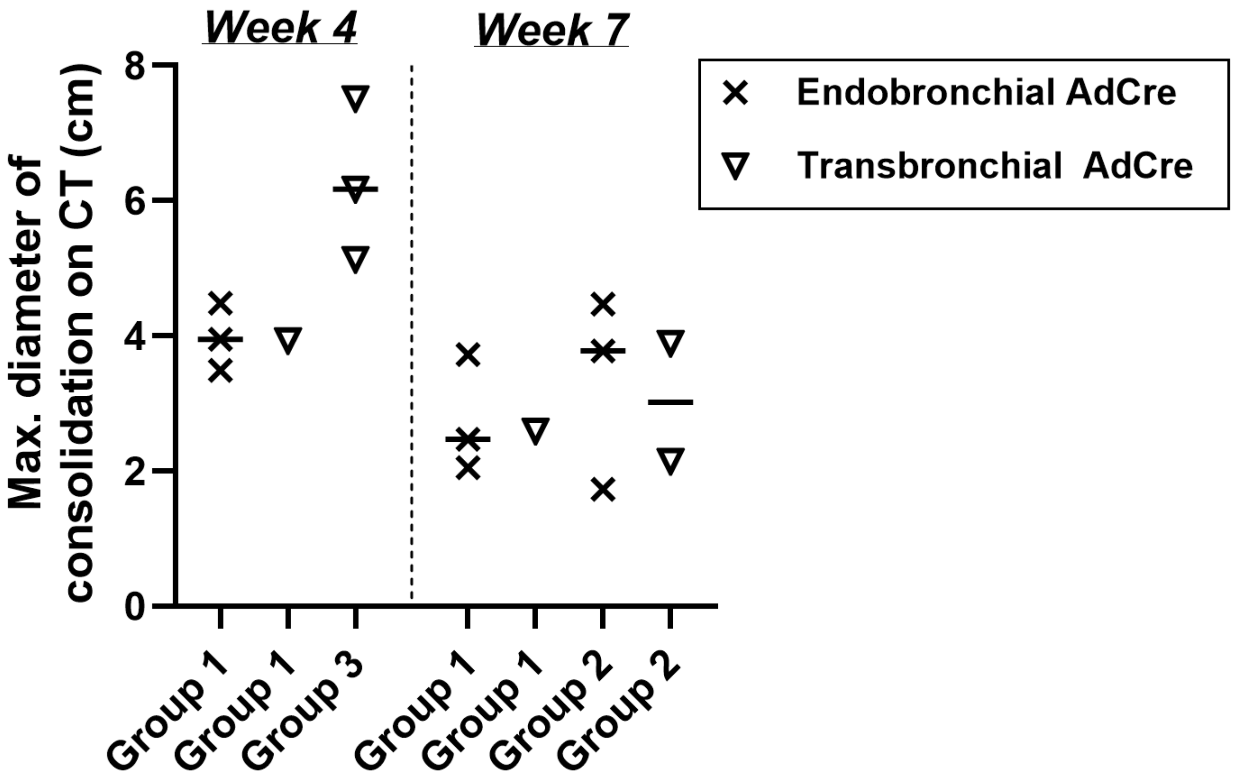

Supplementary Figure 1: Comparison of different groups regarding maximum tumor dimensions measured on contrast-enhanced CT imaging at the same timepoints (week 4 and week 7). At week 4, group 3 (transbronchial) did show larger maximum tumor diameters compared to group 1 (endobronchial), yet not reaching the level of significance (p=0.100; Mann-Whitney test). At week 7, no significant differences were observed between the groups (p=0.807; Kruskal-Wallis test).

SUPPLEMENTARY FIGURE 2

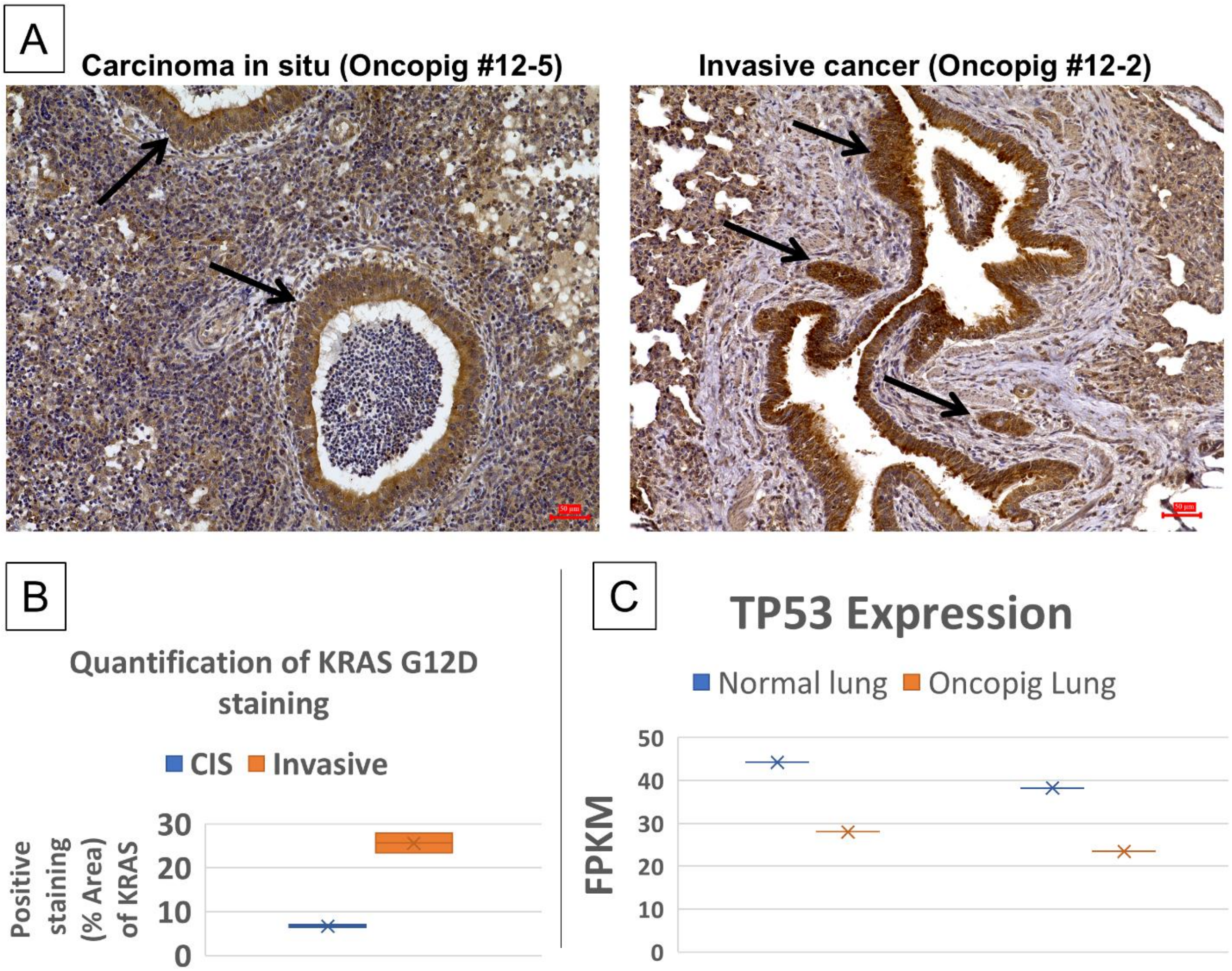

Supplementary Figure 2: **A:** KRAS G12D expression determined with immunohistochemistry in Oncopig lung carcinoma in situ (CIS) showing an epithelial lining composed of multiple layers of atypical cells with intact basement membrane (arrows) [left panel, Oncopig #12-5 (group 2)] and invasive lung cancer (arrows) [right panel, Oncopig #12-2 (group 2)] post bronchoscopic AdCre injection (Bar, 50 μm). **B:** Expression quantification of KRAS G12D immunohistochemistry staining intensity comparing CIS versus invasive cancer (n=3, respectively). **C:** TP53 gene expression in normal lung versus Oncopig lung. (FPKM: fragments per kilobase of transcript per million mapped reads).
